# A Cerebrovascular Incident Secondary to Extensive Aortic Arch Atheroma

**DOI:** 10.7759/cureus.28954

**Published:** 2022-09-08

**Authors:** Faisal A Alghamdi, Saud A AlShehri, Nisreen H Maghraby, Mustafa Shaib, Dunya Alfaraj

**Affiliations:** 1 College of Medicine, Imam Abdulrahman Bin Faisal University, Dammam, SAU; 2 Emergency Department, Imam Abdulrahman Bin Faisal University, King Fahad University Hospital, Dammam, SAU; 3 Emergency Department, Royal Commission Hospital, Jubail, SAU

**Keywords:** ct angiography, aortic arch atheroma, stroke, atherosclerosis, case report

## Abstract

Plaques can form across different parts of the aorta, from the aortic arch to the thoracic and abdominal aorta. Aortic arch atheroma, however, is highly associated with cerebrovascular insults due to their dislodgement. Although no concise management protocol has been defined for dealing with such presentations, antiplatelet agents and anticoagulants are most frequently used. In this case, we present a 78-year-old male with a known case of diabetes mellitus type 2, hypertension, and dyslipidemia who presented to the emergency department with acute onset of slurred speech. A CT angiography was performed that revealed extensive plaque formations across the aortic arch with a 90% occlusion of the distal left common carotid artery and carotid bifurcation along with 99% stenosis of the internal carotid artery. The patient underwent aspiration thrombectomy and was started on dual antiplatelets but passed away after developing decompensated heart failure.

## Introduction

Strokes have been implicated in affecting approximately 16.9 million people a year worldwide [1]. Atherosclerosis of aortic arches can lead to systematic embolization if dislodged with numerous studies finding a strong association between the two [2,3]. Aortic arch atheroma begins to appear in early adulthood and increases with age, hypercholesterolemia, hypertension, diabetes mellitus, increased homocysteine and fibrinogen, and being of the male sex among others [4,5]. It alone causes increases the odds ratio of having a stroke by more than four, and if mobile, the odds increase by more than 12 [4]. The diagnosis of aortic plaques can be detected by proper utilization of transesophageal echocardiography (TEE) [5]. To date, no specific treatment protocol has been established specifically for aortic arch atheroma. However, a study by Ferrari et al. found that oral anticoagulants can be used in most instances, such as in our case [6].

## Case presentation

A 78-year-old male with a known history of diabetes mellitus type 2, hypertension, and dyslipidemia presented to the hospital’s emergency department complaining of slurred speech that began two hours prior to presentation. He denied having a history of loss of consciousness, weakness, numbness, or headaches. Medications included three anti-hypertensives, analgesics, and vitamins. The patient was not on any anti-platelet medication. Upon admission to the emergency department, vital signs taken were as follows: heart rate was 107 beats per minute, blood pressure was 140/95 mmHg, respiratory rate was 23 per minute, and oxygen saturation was 87% on room air. Neurological examination on admission was insignificant. The cranial nerve examination was normal. The tone was normal, power was symmetrical, and reflexes were normal. No ataxia was noted. 

At the time of admission, laboratory investigations revealed elevated high sensitivity troponin I at 9.47 ng/mL (normal range < 0.029 ng/mL), elevated serum glutamic-oxaloacetic transaminase (SGOT) levels at 122 units per liter (U/L) (normal range from 5-34 U/L), and lastly high lactate dehydrogenase (LDH) 630 U/L (normal range 125-220 U/L). International normalized ratio (INR) and electrolytes were within normal limits. A chest x-ray was performed and showed signs of bilateral pulmonary edema and electrocardiography (ECG) revealed sinus tachycardia with non-specific ST changes. The patient was started on clopidogrel, acetylsalicylic acid, furosemide and enoxaparin.

**Figure 1:**
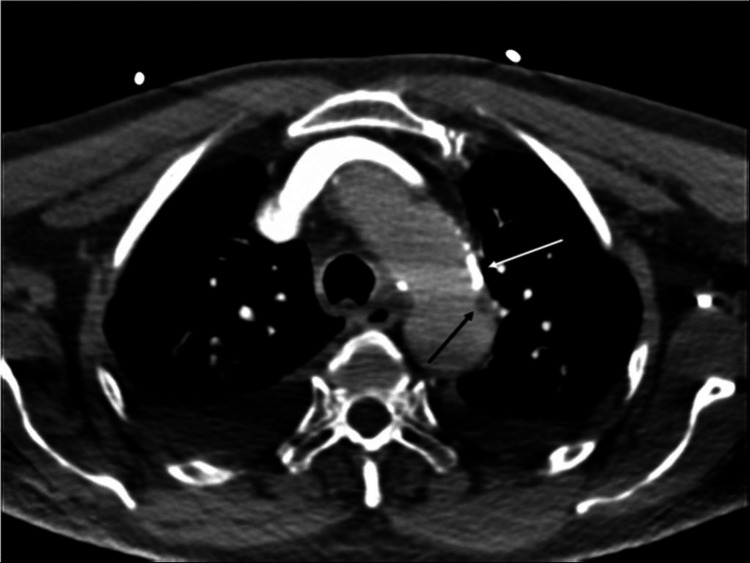
Abdominal computed tomography, transverse section showing the aorta A transverse section of an abdominal computed tomography with contrast in which the white arrow represents calcified atherosclerotic changes while the black arrow represents soft atheroma or noncalcified changes in the aortic arch

**Figure 2:**
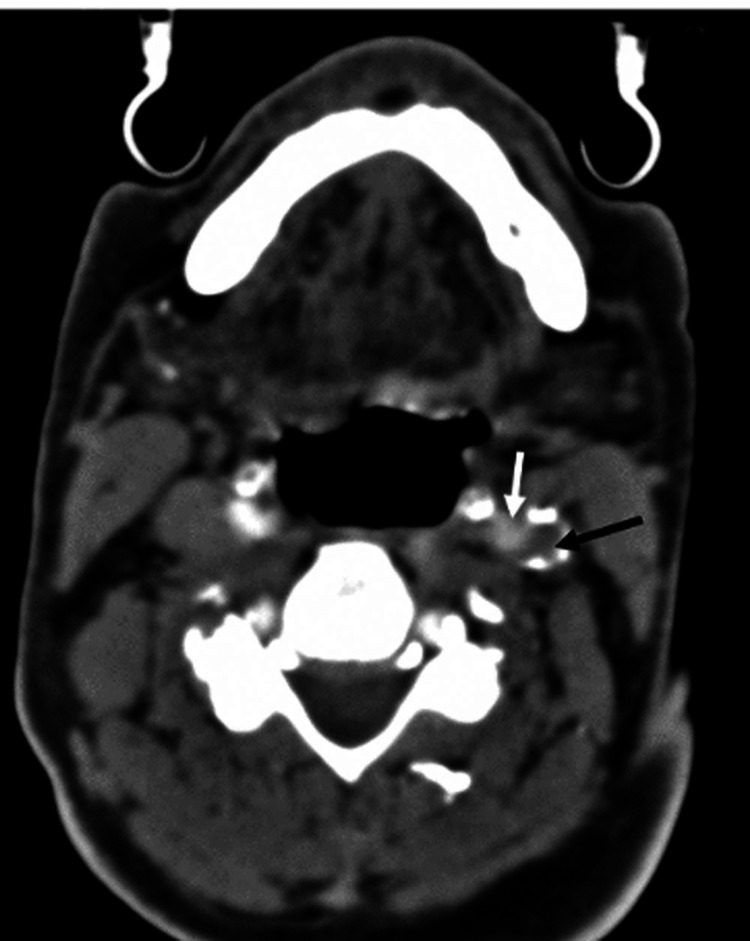
Axial computed tomography of the carotids Axial computed tomography of the carotid revealed opacification and almost complete occlusion of both the external carotid represented by the white arrow, the internal carotid by the black arrow.

**Figure 3:**
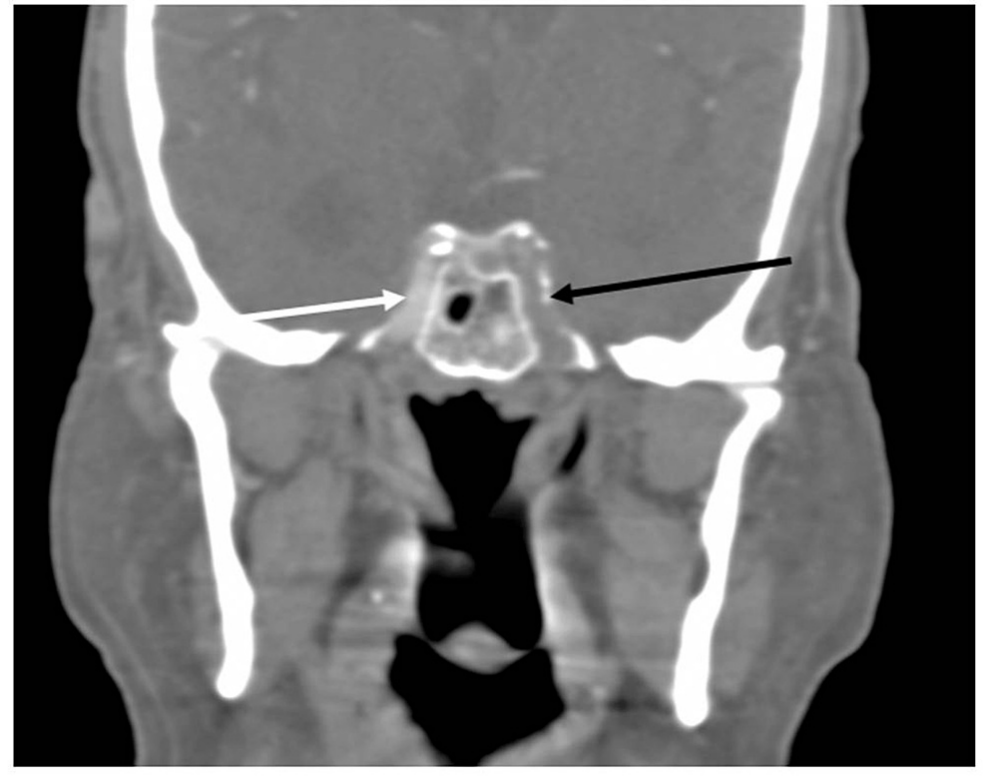
Computed tomography angiography at the level of the cavernous portion, coronal view Computed tomography angiography at the level of the cavernous portion coronal view demonstrates total occlusion of left internal carotid artery (black arrow) while the right internal carotid artery (white arrow) is opacified.

Plain computed tomography (CT) of the head, computed tomography angiography (CTA) of the head, and computed tomography perfusion were ordered after admission of the patient as part of the hospital's protocol. CTA revealed extensive atherosclerotic changes in the aortic arch with non-calcified athermanous plaque (Figure 1). There was also severe stenosis of the distal left common carotid artery and carotid bifurcation exceeding 90% stenosis (Figure 2). It also revealed a newly developed near-total non-filling defect of contrast of the left internal carotid artery (ICA) at approximately 99% starting just distal to left carotid bifurcation till the origin of the left middle carotid artery (Figure 3).

The patient was then started on dual anti-platelet loading doses immediately. The risks and benefits of acute intervention were discussed with the family who agreed on carotid intervention which involved left ICA stenting and aspiration thrombectomy with residual stenosis of 20%. The patient was electively intubated after the procedure and was kept on dual antiplatelets and strict blood pressure control for the remainder of his stay.

Cardiology and neurology teams were both consulted who had the impression that his symptoms occurred because of a carotid thrombus with hypoperfusion that was precipitated by poor cardiac function and hypotension. 

For the remainder of the patient’s hospital course, he remained in the intensive care unit (ICU) with a Glasgow Coma Scale (GCS) of 8/15 (E2V3M2) baseline. The patient developed acute decompensated heart failure three days after admission. The following day sudden desaturation happened with a drop in the GCS to a 4/15 (E1V1M2). He later died because of all the complications.

## Discussion

Aortic arch atheroma plays a vital role as a source that may lead to the development of cerebral emboli [2,3]. In one study [2], transesophageal echocardiography was done on 331 patients with a known cause of brain infarction to confirm the presence of any buildup of plaques along the wall of the aortic arch. It was confirmed that patients with an aortic wall thickness of more than 4 millimeters (mm) had a high incidence. A study by Davila-Roman and colleagues [7] revealed that among 1200 patients undergoing open-heart surgery, 19.3% of patients had severe atheroma of the ascending aorta (more than 3mm). The prevalence increased with age, rising to 32.6% in patients who were older than 80 years old. 

What makes our case unique is the extensiveness and degree of stenosis in the atherosclerotic plaques found. A CTA performed revealed atherosclerosis of the entire aortic arch, distal left common carotid artery and carotid bifurcation, and the cavernous and supra-clinoid portions of the bilateral internal carotid arteries. Similar presentations can be found in a prospective case-control study on patients admitted with ischemic strokes in which only five out of 33 patients with severe stenosis (more than 70%) of the carotid artery were found to be associated with such extensive plaque formation [2].

The risk factors associated with the progression of aortic arch atheroma include male gender, smoking, previous vascular disease, and chronic diseases such as hypertension, diabetes mellitus, hypercholesterolemia, and dyslipidemia [3]. The patient in our case had all the aforementioned risk factors, however, coagulation profiles and electrolytes done on initial admission were all within normal limits. 

An essential component of the embolic stroke workup is identifying the presence of an atherosclerotic aortic arch. Transesophageal echocardiography has been used as a gold standard imaging modality for a considerable period [5]. It offers higher resolution images of the heart and its great vessel roots. Nevertheless, it has been noticed that TEE is an invasive modality, needs sedation, is less available, not safe for unstable patients, and is personnel dependent. Another modality with a less invasive approach, computed tomography angiography, was compared with TEE in a retrospective study that included 250 cases at a large tertiary stroke center [8]. The results that CTA had greater results in detecting higher grades (3 & 4) atheroma with a specificity of 98.9% and sensitivity of 23.3%. Additionally, it has greater advantages such as rapid, non-invasive, and automated. 

In the presence of ischemic stroke with accompanied aortic arch atheroma, it is recommended by the American Heart Association/American Stroke Association to treat patients using statin and antiplatelet therapy. Owing to the small number of studies conducted regarding antithrombotic therapy, no consensus has yet been done on how to manage such cases. However, the Aortic Arch Related Cerebral Hazard (ARCH) trial found that the group of patients using aspirin plus clopidogrel was associated with decreased occurrence of ischemic stroke [9].

## Conclusions

Embolic events can cause fatal complications such as strokes or transient ischemic attacks for patients having aortic arch atheroma. Quick utilization of imaging modalities such as a transesophageal echocardiogram or computed tomography angiography is of extreme importance to rapidly determine the next step in management. The cornerstone of pharmacological therapy is currently antiplatelet agents such as clopidogrel and aspirin. We recommend that further studies be done on managing cases of aortic arch atheroma both pharmacologically and surgically due to the lack of managerial guidelines.
